# LFR Physically and Genetically Interacts With SWI/SNF Component SWI3B to Regulate Leaf Blade Development in Arabidopsis

**DOI:** 10.3389/fpls.2021.717649

**Published:** 2021-08-11

**Authors:** Xiaowei Lin, Can Yuan, Bonan Zhu, Tingting Yuan, Xiaorong Li, Shan Yuan, Sujuan Cui, Hongtao Zhao

**Affiliations:** ^1^Hebei Key Laboratory of Molecular and Cellular Biology, Key Laboratory of Molecular and Cellular Biology of Ministry of Education, Hebei Collaboration Innovation Center for Cell Signaling and Environmental Adaptation, College of Life Sciences, Hebei Normal University, Shijiazhuang, China; ^2^School of Traditional Chinese Medicine, Tianjin University of Traditional Chinese Medicine, Tianjin, China

**Keywords:** LFR, SWI3B, *FIL*, *IAMT1*, SWI/SNF, leaf, Arabidopsis

## Abstract

Leaves start to develop at the peripheral zone of the shoot apical meristem. Thereafter, symmetric and flattened leaf laminae are formed. These events are simultaneously regulated by auxin, transcription factors, and epigenetic regulatory factors. However, the relationships among these factors are not well known. In this study, we conducted protein-protein interaction assays to show that our previously reported Leaf and Flower Related (LFR) physically interacted with SWI3B, a component of the ATP-dependent chromatin remodeling SWI/SNF complex in Arabidopsis. The results of truncated analysis and transgenic complementation showed that the N-terminal domain (25–60 amino acids) of LFR was necessary for its interaction with SWI3B and was crucial for LFR functions in Arabidopsis leaf development. Genetic results showed that the artificial microRNA knockdown lines of *SWI3B* (*SWI3B-amic*) had a similar upward-curling leaf phenotype with that of *LFR* loss-of-function mutants. ChIP-qPCR assay was conducted to show that LFR and SWI3B co-targeted the promoters of *YABBY1*/*FILAMENTOUS FLOWER* (*YAB1*/*FIL*) and *IAA carboxyl methyltransferase 1* (*IAMT1*), which were misexpressed in *lfr* and *SWI3B-amic* mutants. In addition, the association between LFR and the *FIL* and *IAMT1* loci was partly hampered by the knockdown of *SWI3B*. These data suggest that LFR interacts with the chromatin-remodeling complex component, SWI3B, and influences the transcriptional expression of the important transcription factor, *FIL*, and the auxin metabolism enzyme, *IAMT1*, in flattened leaf lamina development.

## Introduction

Leaves are the main sites of photosynthesis, a process that results in the production of food in plants, which are then consumed by animals. Leaf morphology is an important trait that affects the efficiency of photosynthesis and crop yield. Leaves develop from leaf primordia, which are located in the peripheral zone of the shoot apical meristem (SAM). The polarity of leaf primordia along the adaxial-abaxial, proximal-distal, and medio-lateral axes are first established (McConnell and Barton, 1998; Bowman et al., 2002; Du et al., 2018). Cells that are destined to appear on the adaxial side of the leaf are determined by HD-ZIP III and related transcription factors, while those that are destined to appear on the abaxial side of the leaf are established and maintained by YABBY (YAB) and KANADI (KAN) transcription factors. These adaxial and abaxial cell fate regulators are coordinated by auxin and a transcription factor called ASYMMETRIC LEAVES2 (AS2), which act on flattened leaves during their development (Wu et al., 2008; Jun et al., 2010; Husbands et al., 2015; Manuela and Xu, 2020). However, the epigenetic regulatory mechanisms of these regulators and their effect on leaf development should be elucidated.

In eukaryotes, ATP-dependent chromatin remodeling complexes (CRCs) are a group of crucial epigenetic factors that utilize energy from ATP hydrolysis to influence chromatin or nucleosome conformation and transcriptional gene expression (Vignali et al., 2000; Hargreaves and Crabtree, 2011). As a conserved subfamily of CRCs, the SWITCHING/SUCROSE NON-FERMENTING (SWI/SNF) complex usually contains four conserved core subunits, including Swi2/Snf2 ATPase, Swi3, Snf5, and Swp73/BAF60/CHC. These core subunits are required for the assembly and activity of the SWI/SNF complex (Sudarsanam and Winston, 2000; Yang et al., 2007; Sundaramoorthy and Owen-Hughes, 2020). Several core subunits of the plant Swi2/Snf2 ATPase BRAHMA (BRM)-SWI/SNF complex, such as BRAHMA-interacting proteins 1 (BRIP1) and BRIP2, and bromodomain-containing proteins BRD1, BRD2, and BRD13, have recently been discovered to co-localize and act together with BRM on chromatin to regulate gene expression (Yu et al., 2020, 2021). In the genome of the model plant, *Arabidopsis thaliana*, there are four Swi3 proteins, including SWI3A/3B/3C/3D (Sarnowski et al., 2005). Results of a genetic analysis indicate that these components play essential roles in regulating multiple growth and developmental processes (Sarnowski et al., 2005; Han et al., 2018; Jiang et al., 2019; Yang et al., 2020). SWI3A, SWI3B, and SWI3C proteins interact with one another, whereas SWI3D only interacts with SWI3B (Sarnowski et al., 2005). Additionally, SWI3B interacts with a long non-coding (lnc)RNA-binding protein called INVOLVED IN *DE NOVO* 2 (IDN2) or with histone deacetylase HISTONE DEACETYLASE 6 (HDA6) to maintain non-coding RNA-mediated transcriptional or transposon silencing (Zhu et al., 2013; Yang et al., 2020). Moreover, SWI3C is involved in the regulation of leaf size in Arabidopsis and tomato (Vercruyssen et al., 2014; Zhao et al., 2019). Arabidopsis SWI3C and BRM interact with the transcription factor, TEOSINTE BRANCHED1, CYCLOIDEA, PCF4 (TCP4), to promote cell differentiation in leaves by increasing the transcriptional expression of *ARABIDOPSIS RESPONSE REGULATOR 16* (*ARR16*), an inhibitor of cytokinin response (Efroni et al., 2013). Embryos of the null mutants of *SWI3B* genes exhibited early lethality (Sarnowski et al., 2005), whereas knockdown mutants of *SWI3B* with RNA interference (*SWI3B-RNAi*) resulted in an upward-curling leaf phenotype (Han et al., 2018). The increased transcript level and decreased nucleosome occupation of *IAA carboxyl methyltransferase 1* (*IAMT1*) may explain this defect observed during the development of leaves with *SWI3B-RNAi* (Han et al., 2018). However, the direct targets of SWI3B and its interacting partners in leaf development still need clarification.

The *Leaf and Flower-Related gene* (*LFR*) encodes a nuclear protein with the Armadillo (ARM)-repeat domains (Wang et al., 2009), which are involved in protein–protein interactions (Samuel et al., 2006). Arabidopsis with a loss-of-function mutation in the *LFR* gene exhibit pleiotropic phenotypes during leaf and flower development (Wang et al., 2009, 2012; Lin et al., 2018). LFR has been isolated from tandem affinity-purified protein complexes using SWIP37B (Vercruyssen et al., 2014). It interacts genetically and physically with AS2 to co-repress the transcription expression of *BREVIPEDICELLUS* (*BP*), which influences chromatin configuration during the determination of petiole length, vasculature pattern, and leaf margin development (Lin et al., 2018). However, the interacting partners and downstream targets of LFR during the development of flattened lamina remain largely unknown.

This study aimed to determine the interacting partner of LFR, examine the physical and genetic relationships between LFR and SWI3B during flattened leaf development in Arabidopsis, detect changes in the expression of the *FIL* and *IAMT1* genes in Arabidopsis with single mutant of *lfr* and in those with knock-down mutants of *SW13B*, and investigate the binding peaks of LFR and SW13B in the *FIL* and *IAMT1* promoter regions.

## Materials and Methods

### Plant Growth Conditions

We used *A. thaliana*, the commonly used and well-studied model plant, in this study. All Arabidopsis plants in this study had a Columbia-0 background. The seeds of *lfr-1* and *lfr-2/*+ were previously reported in our laboratory (Wang et al., 2009). *swi3b-2/*+ were previously reported (Sarnowski et al., 2005). Other transgenic plants were obtained in this sturdy by floral infiltration (Clough and Bent, 1998), after successful plasmid constructions described in the next part. The seeds were surface-sterilized with 75% ethanol, stored at 4°C for 3 days, and cultured on Murashige and Skoog (MS) medium containing 1% sucrose (pH 5.7). After 10 days of growth, the seedlings were transplanted into soil and grown in a greenhouse under a 16-h light/8-h dark photoperiod at 22°C.

### Plasmid Constructions

For the binary vectors for the transgenic complementation and genetic analysis, the coding sequences of the full or truncated *LFR*, *SWI3B*, and *FIL* were amplified with specific primers (Supplementary Table 1) using the plasmid pTR5 (for *LFR*) (Wang et al., 2009) or cDNA (for *SWI3B* and *FIL*) as the template. The amplified fragment was digested using an appropriate restriction endonuclease and inserted into p*CAMBIA1300 35S:3FLAG* to obtain p*35S:LFR* (full length or truncated)-*3FLAG*, *35S:SWI3B-3FLAG*, and *35S:FIL-3FLAG*.

The yeast two-hybrid (Y2H) GAL4 system bait/prey plasmid, which had a coding sequence of full or truncated LFR or SWI3B were separately constructed. Briefly, the coding sequences of the full or truncated *LFR* or *SWI3B* were amplified with specific primers (Supplementary Table 1) using the plasmid, pTR5 (Wang et al., 2009), and cDNA as a template. The amplified fragment was digested using an appropriate restriction endonuclease and inserted into prey pGADT7/bait pGBKT7 to obtain pGADT7/pGBKT7-LFR (full length or truncated), pGADT7/pGBKT7-SWI3B (full length or truncated).

In bimolecular fluorescence complementation (BiFC) experiments, full-length CDS of SWI3B with a stop codon was amplified via polymerase chain reaction (PCR) using the Arabidopsis cDNA as a template and cloned into pENTRY/D/SD-TOPO. These genes were then introduced into pxnYFPGW via the LR reaction. The N terminal part of nYFP-AS2 and the C terminal part of CFP-LFR (cCFP-LFR) plasmids were reported in our previous study (Lin et al., 2018). The specific primers used for plasmid construction are listed in Supplementary Table 1.

For artificial miRNA construction of *SWI3B*, artificial miRNA site selection, primers, and specific construction procedures were carried out according to the description on the Web of MicroRNA Designer platform (WMD)^1^. The artificial miRNA precursors, *mic1* and *mic2*, were amplified via PCR using specific I-IV primers (Supplementary Table 1) and plasmid pRS300 as template. The artificial miRNA precursors were digested with *Spe*I and *Kpn*I and inserted into the pMDC32 binary vector. All the constructs were identified via DNA sequencing.

### Reverse Transcription-Polymerase Chain Reaction (RT-PCR) and Quantitative Real-Time Polymerase Chain Reaction (qRT-PCR)

For RT-PCR, total RNA was isolated using the RNAiso Plus reagent (TaKaRa)^2^. First-strand cDNA was synthesized using 500 ng of total RNA and the one-step RT-PCR kit (TaKaRa). PCR fragments were subsequently amplified using their corresponding primers (Supplementary Table 1), analyzed via agarose gel electrophoresis, and stained with the Goldview^TM^ nucleic acid stain (SBS Genetech Co., Ltd., China).

We then conducted qRT-PCR. Total RNA (500 ng) isolated from the leaves was reverse transcribed using the SYBR PrimeScript^TM^ RT-PCR Kit (TaKaRa) to synthesize cDNA. PCR amplification was performed using the SYBR^®^ Premix Ex Taq^TM^ kit (TaKaRa). The gene-specific primers used are listed in Supplementary Table 1 for the qRT-PCR reactions. *eIF4A1* was used as an internal control.

### Total Protein Extracts and Western Blot Assay

Total proteins were extracted from 1 g of 14-day-old seedlings and dissolved in sample buffer (50 mM Na_2_HPO_4_/NaH_2_PO_4_, pH 7.4; 150 mM NaCl; 1% Triton X-100; 15% glycerol; 1 mM PMSF; and 1 × cocktail). Isolated proteins were identified using 10% sodium dodecyl (lauryl) sulfate-polyacrylamide gel electrophoresis (SDS-PAGE) gel. They were transferred onto polyvinylidene difluoride (PVDF) membranes using a semi-dry electroblotter (Bio-Rad). The PVDF membranes were probed with anti-FLAG (Sigma), anti-H3 (Agrisera), anti-LFR (Lin et al., 2018), anti-SWI3B (Sarnowski et al., 2002) or anti-tubulin antibody (Sigma). Goat anti-rabbit or anti-mouse IgG secondary antibodies were used for immunodetection.

### Co-immunoprecipitation (co-IP) Assay

Approximately 4 g of 10-day-old *Arabidopsis* seedlings were used for immunoprecipitation experiments. The seedlings were extracted and added to a 4 mL protein solution buffer (50 mM Na_2_HPO_4_/NaH_2_PO_4_, pH 7.4; 150 mM NaCl; 1% Triton X-100; 15% glycerol; 1 mM PMSF; and protease inhibitor cocktail from Roche). The extracts were centrifuged at 17,000 × *g* for 10 min at 4°C. The supernatant proteins were then incubated with 40 μL of anti-FLAG M2 agarose beads (Sigma, Cat. # M8823) for 1 h at 4°C. After incubation, the beads were collected by centrifugation and washed three to five times with 1 mL wash buffer (50 mM Na_2_HPO_4_/NaH_2_PO4, pH 7.4; 150 mM NaCl; 0.1% Triton X-100; 10% glycerol; 1 mM PMSF; and protease inhibitor cocktail from Roche). The antigen-antibody complex was boiled in Laemmli SDS-PAGE buffer (125 mM Tris–HCl, pH 6.8; 4% SDS; 20% glycerol; 2% mercaptoethanol; and 0.001% bromophenol blue), separated on a 12% SDS-PAGE gel, and transferred onto a PVDF membrane. Proteins immunoprecipitated with the anti-FLAG antibodies were probed with anti-LFR polyclonal antibody, which was previously prepared in our lab (Gao et al., 2008), or with anti-SWI3B antibody reported previously (Sarnowski et al., 2002). Secondary antibody (goat anti-rabbit IgG) was used for immunodetection. The SuperSignal West Femto System (Pierce) was used for signal detection.

### Yeast Two-Hybrid (Y2H) Analysis

The Y2H screening of cDNA library derived from 9-day-old seedlings of Arabidopsis was performed following the manufacturer’s instructions (Matchmaker GAL4 Two-Hybrid System 3 & Libraries User Manual Clontech Laboratories). We conducted a Y2H assay. Briefly, the bait plasmid, pGBKT7, or prey plasmid, pGADT7, with full-length or truncated LFR or SWI3B were co-transformed into AH109. The co-transformed colonies were selected to grow on a selective medium that lacked leucine and tryptophan (SD/-L-W). A growth assay was then conducted, in which the physical interaction between different pair of proteins was tested on selective medium that lacked leucine, tryptophan, adenine, and histidine (SD/-L-W-A-H). Liquid β-galactosidase (β-Gal) assays, with o-nitrophenyl β-D-galactopyranoside (ONPG) (Sigma) as a substrate, were measured as described in the manufacture’s handbook (Clontech Yeast Protocols Handbook). One unit of β-galactosidase activity was defined as the amount in which hydrolysis of 1 μ mol of ONPG to o-nitrophenol and D-galactose per min per cell occurred.

### Bimolecular Fluorescence Complementation (BiFC) Assay

The BiFC assay was performed as previously described (Ou et al., 2011). The plasmids were separately introduced into Agrobacterium GV3101 and co-infiltrated into the young flattened leaf blade of *Nicotiana benthamiana*. After incubation for approximately 48 h, images were captured using a Zeiss LSM 710 confocal microscope. Green fluorescent protein (GFP) and 4, 6-diamidino-2-pheylindole (DAPI) signals were examined at 488 and 405 nm, respectively.

### Chromatin Immunoprecipitation (ChIP) Assay

The chromatin immunoprecipitation and qPCR (ChIP-qPCR) assay was carried out as previously described (Yamaguchi et al., 2014) with minor modifications. Approximately 0.3–0.6 g of seedlings or the first to third rosette leaves of the 14-day-old seedlings were crosslinked with 1% formaldehyde and fully ground in liquid nitrogen. Chromatin was isolated and cut into approximately 500 bp DNA fragments via sonication. The chromatin suspension was incubated for 2 h with 50 μL of magnetic protein G beads (Invitrogen, Cat. # 10004D), 5 μg of anti-FLAG antibody (Sigma Cat. # F3165), 5 μg of anti-trimethyl-histone H3 (Lys4) (Millipore Cat. # 07-473), or 2 μL of anti-LFR rabbit polyclonal antiserum from our laboratory (Gao et al., 2008). Pre-immune serum was used as the control. DNA was isolated using the DNA purification kit (Qiagen, Cat. # 28104) and used as the template of primers listed in Supplementary Table 1 in real-time qPCR.

## Results

### LFR Physically Interacts With SWI3B in Yeast and Plant

**FIGURE 1:**
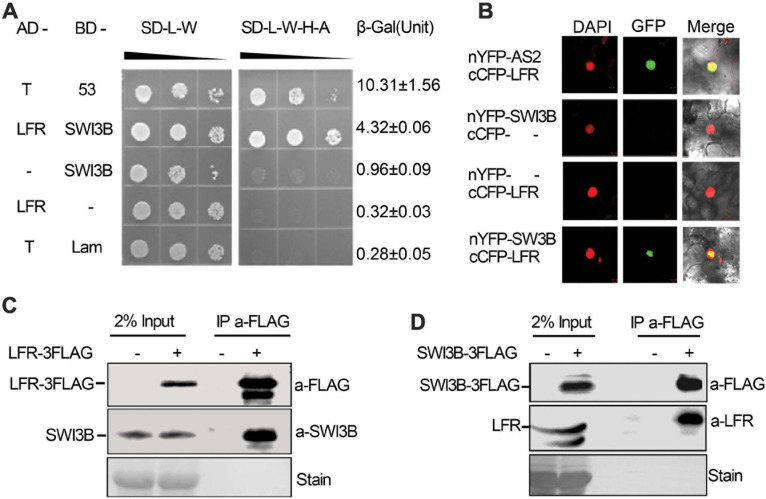
Leaf and flower related (LFR) interacts with SWI3B in yeast and *planta*. **(A)** The growth assay and quantitative β-galactosidase (β-Gal) activity assays showing that AD-LFR interacts with BD-SWI3B in the Y2H assay. The growth experiment was performed on selective medium (SD/-L-W and SD/-L-W-A-H) after gradient dilution (10^– 1^, 10^– 2^, and 10^– 3^) as indicated by black triangles. The AD-T/BD-p53 and AD-T/BD-Lam co-transformed yeast colonies were used as the positive and negative control, respectively. In quantitative β-Gal activity assays, data are mean ± standard error from three independent experiments. **(B)** BiFC assay showing that cCFP-LFR interacts with nYFP-SWI3B in transiently transformed epidermal cells of tobacco leaf (22 of 114 cells had GFP signal). DAPI signal indicates nucleus. GFP signal shows interaction. Merge means overlay of DAPI and GFP fluorescence signals. cCFP-LFR/nYFP-AS2 serves as a positive interaction control; - no protein fusion. **(C,D)** Co-IP assay identifies LFR-3FAG and SWI3B co-exist in *35S:LFR-3FLAG/lfr-1* transgenic rescue line **(C)**, and SWI3B-3FAG and LFR co-exist the same complex in *35S:SWI3B-3FLAG/swi3b-2* transgenic rescue line **(D)**. Total protein extracts were derived from 14-day-old seedlings of Col-0 (–) or transgenic rescue line (+). Anti-FLAG antibody beads were used to immunoprecipitate (IP a-FLAG). In western blot, anti-FLAG (a-FLAG) or anti-SWI3B (a-SWI3B) or anti-LFR (a-LFR) antibody was used to detect LFR-3FLAG/SWI3B-3FLAG or native SWI3B or LFR, respectively. Ponceau stain (stain) serves as the loading control.

In our previous study, the transcription factor *AS2* was identified as an *LFR*-interacting protein through genetic screening, which explained the molecular mechanism of the developmental of defects in petiole length, vasculature pattern, and leaf margin except for the leaf blade upward-curling defects in *lfr* mutants (Lin et al., 2018). To further elucidate the molecular mechanisms of the functions of LFR in flattened leaf blades, we screened the cDNA library of 9-day-old seedlings of Arabidopsis to identify possible LFR-interacting proteins by Y2H. Since the full-length LFR had transcriptional self-activation activity in the yeast AH109 strain (Yuan et al., 2012), LFRΔC2 (1–310 amino acids), which has no transcriptional self-activation activity, was used as a bait to screen the cDNA library. A total of 79 positive in-frame proteins were identified (Supplementary Table 2). Sequencing analysis showed that one positive colony contained the full-length coding sequence of *SWI3B*, which was a component of the SWI/SNF complex in Arabidopsis. To further verify the interaction between LFR and SWI3B, the Y2H assay was performed using the full-length LFR, which was fused with AD and BD-SWI3B. Yeast AH109 colonies, which were co-transformed with BD-SWI3B and AD-LFR, grew well on selective medium and had a much higher β-Gal activity than the negative control; however, BD-SWI3B and AD-LFR had no self-activation (Figure 1A). These findings indicate that LFR interacts directly with SWI3B in yeast.

To further confirm the interaction between LFR and SWI3B in plant cells, BiFC assay was performed in *N. benthamiana* leaves. We observed GFP signals in cells that were co-transformed with *cCFP-LFR/nYFP-AS2* plasmid as a positive control (Lin et al., 2018), but GFP signals were rarely observed in nuclei that were co-transformed with cCFP empty vectors, nYFP-SWI3B or nYFP empty vectors, and cCFP-LFR (Figure 1B). Under these experimental conditions, GFP signals were observed in the nuclei of epidermal cells co-transformed with cCFP-LFR and nYFP-SWI3B (Figure 1B). Therefore, the results of the BiFC assay show that LFR interacts with SWI3B in plant.

To further test whether LFR interacts with SWI3B in Arabidopsis, we prepared transgenic complementary lines of *35S:LFR-3FLAG/lfr-1* and *35S:SWI3B-3FLAG*/*swi3b-2* (Supplementary Figures 1, 2). We performed a co-immunoprecipitation (co-IP) assay using total protein extracts isolated from LFR-3FLAG or SWI3B-3FLAG transgenic seedlings. Anti-FLAG antibody-coated beads were used to immunoprecipitate LFR-3FLAG and its associated proteins. We then used anti-SWI3B antibodies to detect endogenous SWI3B proteins, which were only detected in *35S:LFR-3FLAG/lfr-1* transgenic rescue plants but not in their wild-type counterparts (Figure 1C). In the co-IP assay in *35S:SWI3B-3FLAG* transgenic rescue plants, SWI3B-3FLAG was also specifically co-precipitated with endogenous LFR (Figure 1D). These co-IP data indicate that LFR and SWI3B co-exist in the same complex in Arabidopsis.

There are four SWI3 proteins in the genome of Arabidopsis: SWI3A, SWI3B, SWI3C, and SWI3D (Sarnowski et al., 2005). We examined the interactions between the following pairs in yeast: LFR and SWI3A; LFR and SWI3C; and LFR and SWI3D. Since BD-SWI3C and BD-SWI3D had transcriptional self-activation activity, yeast AH109 colonies co-transformed with BD-LFRΔC2 and AD-SWI3C/SWI3D were tested via a growth assay. The Y2H results show that in yeast, LFR interacts with SWI3A but not with SWI3C and SWI3D (Supplementary Figure 3).

### The N-Terminal Domain of LFR Is Essential for the Interaction Between LFR and SWI3B

**FIGURE 2:**
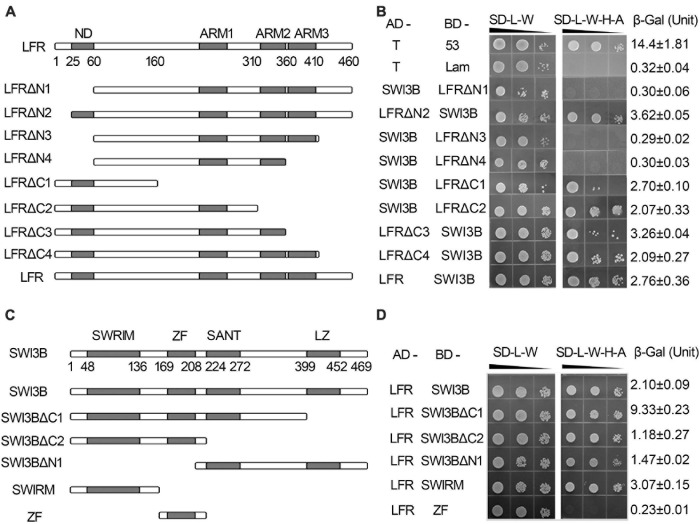
The interacting domain analysis of LFR and SWI3B. **(A,C)** Schematic of full-length and truncated LFR **(A)** and SWI3B **(C)**. **(B,D)** The interacting domain of LFR with full-length SWI3B **(B)** and interacting domain of SWI3B with full-length LFR **(D)** used in Y2H growth and quantitative β-Gal activity assay. Yeast colonies were tested for growth assay on SD-L-W or SD-L-W-H-A after gradient dilution (10^– 1^, 10^– 2^, and 10^– 3^) as indicated by black triangles. Numbers on the right represent the mean ± standard error of three biological replicates of the β-Gal activity.

Our previous report indicated that the C terminus of LFR had three predicted protein–protein interaction ARM-repeat domains responsible for the self-activation activity of BD-LFR in yeast (Yuan et al., 2012). Here, a series of truncated LFR without self-activation activity was used to further identify the interaction domain between LFR and SWI3B in yeast. The truncated LFR without the ARM domains, including LFRΔC1-C4, interacted with SWI3B (Figures 2A,B, Supplementary Figure 4). Upon deletion of the N-terminal domain (ND) of LFR (25–60 amino acids), the interaction between LFR and SWI3B was abolished (Figures 2A,B and Supplementary Figure 4), indicating that the ND motif of LFR was essential for its interaction with SWI3B.

We also determined the specific region of SWI3B that was involved in the interaction with LFR. The SWI3B protein included the SWIRM domain, zinc finger (ZF, homologous with the ZF domain of SWI3D), SANT, and leucine zipper (LZ) domain (Bateman et al., 1999; Sarnowski et al., 2005). The results of the growth assay reveal that all combinations, except for BD-ZF and AD-LFR, can activate the reporter genes (Figures 2C,D and Supplementary Figure 4B). These results suggest that SWIRM, SANT, and LZ but not the ZF domain of SWI3B were able to interact with LFR.

### The Biological Function Analysis of Truncated LFR by Transgenic Rescue Assay

**FIGURE 3:**
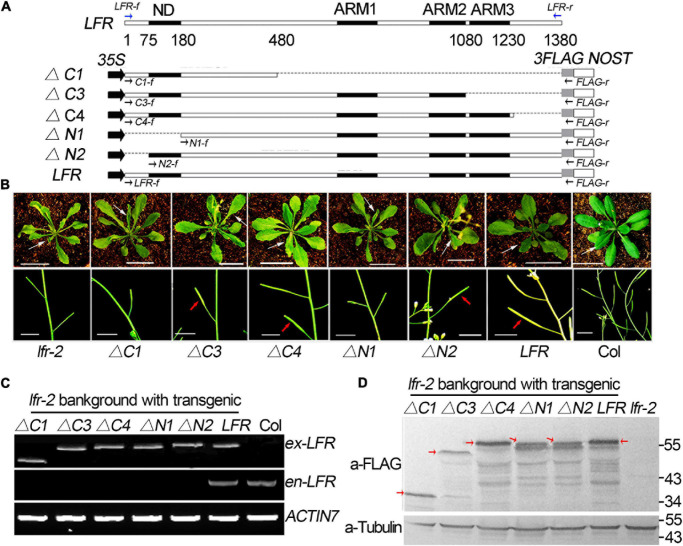
Transgenic rescue analysis of truncated LFR in *lfr-2* null mutant. **(A)** Schematic map of constructs used in the transgenic rescue analysis. *LFR* indicated full length coding sequence (CDS) of LFR (1380 bp). *35S*, *NOST*, and *3FLAG* represented the *CaMV 35S* promoter, the terminator sequence of the *NOS* gene, and the tag, respectively. The numbers below LFR gene structure showed the exact nucleic acids of the LFR CDS coding for the corresponding protein domains. Full length and truncated LFR (*LFR*, Δ*C1*, Δ*C3*, Δ*C4*, Δ*N1*, and Δ*N2*) were inserted into the *35S:3FLAG* vector and transformed into *lfr-2*. The blue and black arrows represent primers used for endogenous *LFR* (*en-LFR*) and different lengths of exogenous *LFR* (*ex-LFR*), respectively, used in RT-PCR in panel **(C)**. **(B)** The leaf (from 35-day-old plants, upper panel) and siliques (from 50-day-old plants, bottom panel) of Col, *lfr-2*, and different transgenic lines in the *lfr-2* background. The white arrowhead pointed to the leaves from a similar position of different genotypes. The red arrowhead pointed to the elongated siliques with seeds. Bar = 2 cm in upper panel, Bar = 1 cm in bottom panel. **(C)** RT-PCR analysis of endogenous (*en-*) and exogenous (*ex-*) full length or truncated *LFR* in different genotypes with the primers showed in panel **(A)**. *ACTIN7* was used as the loading control. **(D)** Western blot with anti-FLAG monoclonal antibody (a-FLAG) or anti-Tubulin (a-Tubulin) in Col and transgenic lines. The red arrows represented the corresponding truncated or full LFR-FLAG fusion proteins. The signal underlying LFR-FLAG fusion protein is caused by the degradation of LFR-FLAG fusion proteins. Tubulin was used as the internal loading control.

To explore the importance of the ND motif for the biological function of LFR in plant development, we fused *LFR*Δ*N1* and *LFR*Δ*N2* with *3FLAG* driven by the *CaMV 35S* promoter to obtain *35S:LFR*Δ*N1-3FLAG* and *35S:LFR*Δ*N2-3FLAG*, respectively. We then transformed them into the *lfr-2* background (Figure 3A). As a control, *35S*:*LFR-3FLAG* completely rescued the upward-curling leaf and sterile defects of *lfr-2*. Four transgenic lines of *35S:LFR*Δ*N2-3FLAG/lfr-2* without the N-terminal 1–25 amino acids could also recover the defects of *lfr-2* in leaf and silique development. However, the *35S*:*LFR*Δ*N1-3FLAG* construct, with further deletion of the ND region of LFR, could not rescue any phenotype of *lfr-2* (Figure 3B). To ensure that *LFR*Δ*N1-3FLAG* was normally expressed, we carried out RT-PCR and Western blotting and found that it could be expressed normally at both the RNA and protein levels (Figures 3C,D). These data demonstrate that the ND motif responsible for the LFR-SWI3B interaction is crucial for the full biological function of LFR in Arabidopsis.

Meanwhile, we constructed a truncated LFR without one or more ARM domains, including *LFR*Δ*C1*, *LFR*Δ*C3*, and *LFR*Δ*C4*, into the *35S:3FLAG* vector (Figure 3A). We then introduced them into the *lfr-2* background. Three transgenic lines of *35S:LFR*Δ*C3-3FLAG*/*lfr-2* and six transgenic lines of *35S:LFR*Δ*C4-3FLAG/lfr-2* were observed to partially rescue the leaf and silique phenotypes of *lfr-2* (Figure 3B). However, the transgenic homozygous lines expressing *35S:LFR*Δ*C1-3FLAG* without the ARM 1–3 domain could not rescue any phenotype of *lfr-2* (Figure 3B). In addition, we conducted RT-PCR and Western blotting and found that *LFR*Δ*C1-3FLAG* was normally expressed at both the RNA and protein levels (Figures 3C,D).

Together, these transgenic complementary data suggest that both the ND and ARM domains are crucial for the biological function of LFR in Arabidopsis.

### *LFR* Genetically Interacts With *SWI3B* During Leaf Blade Development

**FIGURE 4:**
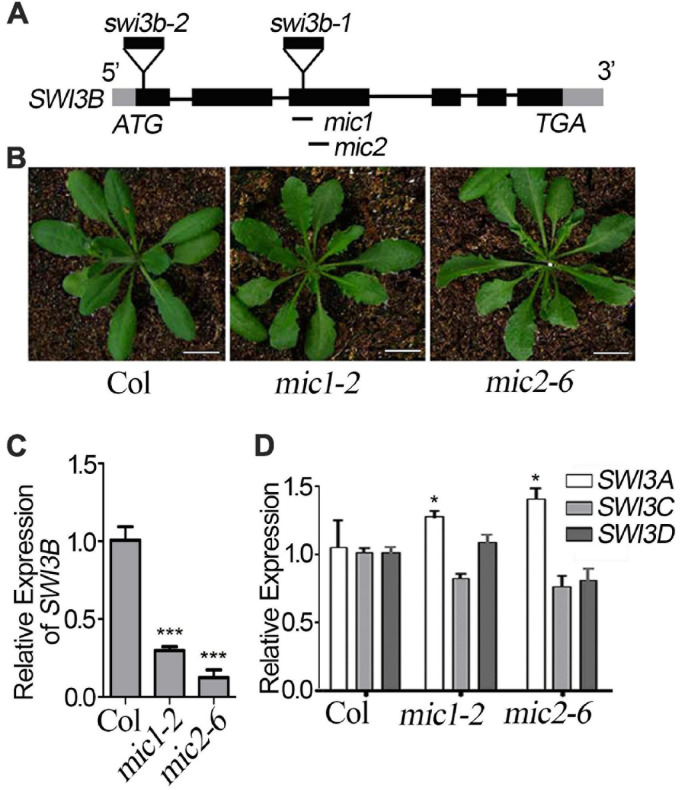
Transgenic lines of artificial miRNA-mediated knockdown of *SWI3B*. **(A)** Schematic gene model and mutants of *SWI3B*. *swi3b-1* and *swi3b-2* are T-DNA insertion mutants reported previously (Sarnowski et al., 2005). The target sites of two artificial microRNA of *SWI3B* (*mic1*: 594–614 bp and *mic2*: 753–773 bp) were indicated by black lines below the gene structure. **(B)** The phenotypes of T1 transgenic lines of artificial microRNA of *SWI3B* (*mic1* and *mic2*). The appearance of 35-day-old plants from different genotypes as indicated. Scale bars = 1 cm. **(C,D)** SWI3B genes expression level **(C)** and *SWI3A*, *SWI3C*, and *SWI3D* genes expression level **(D)** analyzed by quantitative reverse transcription-PCR (qRT-PCR). The RNA was extracted from the 35-day-old Col and *SWI3B-amic* T1 lines. *eIF4A1* was used as an internal control. Bars indicate the means ± SE of three independent biological repeats. Significant statistical differences were tested using Student’s *t*-test (***P < 0.001, *P < 0.05).

To detect the genetic relationship between *LFR* and *SWI3B*, we created the knock-down mutants of *SWI3B* using artificial microRNA to produce *SWI3B-amic* because the null mutants of *SWI3B* (*swi3b-1* and *swi3b-2*) were embryo-lethal (Sarnowski et al., 2005). We chose two sites, *mic1* and *mic2* (short for *SWI3B-amic1* and *SWI3B-amic2*), for the design of *SWI3B* artificial microRNA (Figure 4A). We obtained four independent transgenic homozygous lines for *mic1* and ten for *mic2* in the wild-type background. Transgenic *mic1-2* and *mic2-6* lines, which had low transcript level of *SWI3B*, had upward-curling leaves compared to the wild type (Figures 4B,C). To detect whether *mic1-2* and *mic2-6* specifically targeted *SWI3B*, we measured the expression levels of *SWI3B* homologous genes, which included *SWI3A*, *SWI3C*, and *SWI3D*. The transcript levels of *SWI3C* and *SWI3D* had no obvious changes; however, a slight increase in the *SWI3A* transcript was noted (Figure 4D). These data indicate that *mic1-2* and *mic2-6* specifically target *SWI3B* and result in an upward-curling leaf phenotype, which is similar to that of plants with *LFR* loss-of-function mutation (Wang et al., 2009; Figure 3B).

**FIGURE 5:**
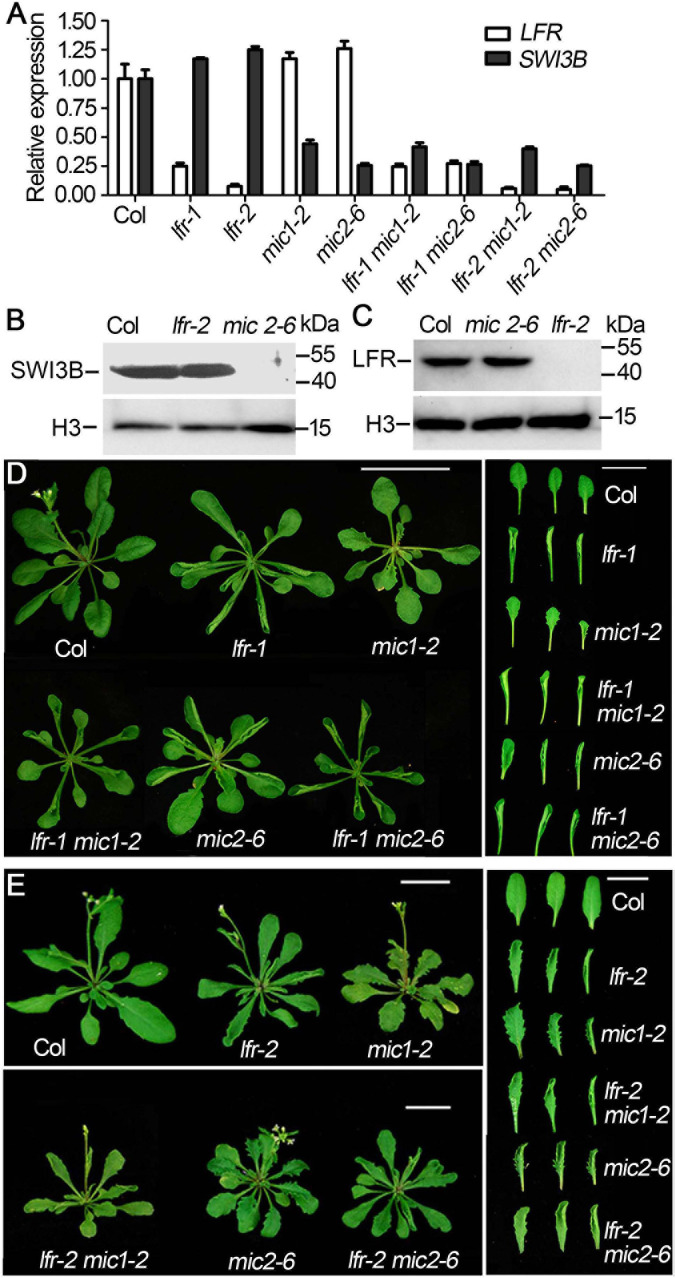
The genetic interaction between *lfr* and *SWI3B-amic* in leaf development. **(A)** qRT-PCR analysis of *SWI3B* and *LFR* transcript in 35-day-old Col and *lfr-1*, *mic1-2*, and *mic2-6* single or double mutant plants. The total RNA was isolated from the 7^th^ and 8^th^ rosette leaves of 35-day-old Col or various mutants. *eIF4A1* was used as an internal control. **(B,C)** Western blot analysis of SWI3B **(B)** and LFR **(C)** protein level in from different genotypes as indicated, and the H3 served as a loading control. The total protein was extracted from the 14-day-old seedlings of Col and different mutants. **(D,E)** Genetic interaction between *SWI3B-amic* and *lfr-1* were assessed at 35 days of age **(D)** and *lfr-2* at 40 days of age **(E)**. The phenotype of whole plants (left) and the representative 8^th^–10^th^ rosette leaves (right) from different genotypes as indicated. Scale bars = 2 cm in panels **(D,E)**.

We then obtained double mutants of *lfr-1/2 mic1-2* or *lfr-1/2 mic2-6* by genetic crossing. qRT-PCR data showed that the double mutants had significantly reduced the expression of *LFR* and *SWI3B* (Figure 5A). The transcript and protein levels of *SWI3B* did not change significantly in *lfr* mutants (Figures 5A,B). Meanwhile, we did not detect obvious changes in LFR at the RNA and protein levels in *mic1-2* and *mic2-6* (Figures 5A,C). Therefore, these data indicate that LFR and SWI3B do not regulate each other at the transcriptional and protein levels. We then analyzed the phenotypic characteristics of *lfr-1*, *lfr-2*, *mic1-2*, and *mic2-6* single and double mutants. *lfr-1*, *lfr-2*, *mic1-2*, and *mic2-6* all displayed upward-curling leaves and had a sawtooth appearance at the margin of the leaf blade (Figures 5D,E). The upward-curling leaf phenotype in double mutant *lfr-1 mic1-2* or *lfr-1 mic2-6* was a little stronger that of the single mutant (Figure 5D). The same results were observed in the *lfr-2 mic1-2* or *lfr-2 mic2-6* double mutants (Figure 5E).

Taken together, these genetic data suggest that *LFR* and *SWI3B* may have overlapping functions in the regulation of flattened leaf blade development.

### The Differentially Expressed Genes in *lfr-2* and *SWI3B-amic* Leaves

**FIGURE 6:**
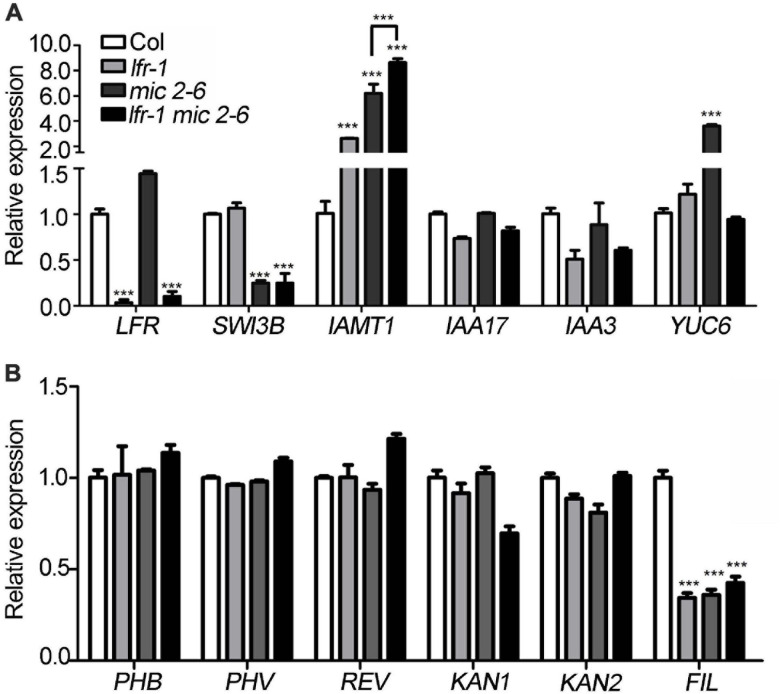
Differentially expressed genes in *lfr-1*, *mic2-6* single and double mutants. **(A,B)** The qRT-PCR data for tested the transcript level of auxin metabolism and synthesis genes **(A)** and leaf polarity genes **(B)** in different backgrounds as indicated. The total RNA was isolated from the 7th and 8th rosette leaves of 35-day-old Col or various mutants. Transcript levels were normalized to loading control gene *eIF4A1*. Bars indicate the means ± SE of three independent biological repeats. Significant statistical differences were tested using Student’s *t*-test (***P < 0.001).

To identify differentially expressed genes in *lfr-2* and *SWI3B-amic* leaves, we examined the transcript levels of genes encoding the major transcription factors involved in the control leaf polarity, including *HD-ZIP III* (*PHABULOSA*, *PHB*; *PHAVOLULA*, *PHV*; *REVOLUTA*, *REV*) for adaxial cell fate determination, and *YAB1* (*FIL*) and *KAN* (*KAN1* and *KAN2*) family genes for abaxial cell fate establishment. And the *ASYMMETRIC LEAVES2* (*AS2*) and Knotted in *A. thaliana* (*KNAT*) and some other genes which were already tested previously in our study (Lin et al., 2018) were not included here. We also examined several genes related to auxin metabolism and synthesis, including *IAMT1*, *INDOLE-3-ACETIC ACID INDUCIBLE 17* (*IAA17*), *IAA3*, and *YUCCA* (*YUC6*) in the wild type, *lfr-2*, and *SWI3B-amic* single and double mutants. There was a significant increase in the expression levels of *IAMT1* in *lfr-2* and *SWI3B-amic* single mutants and even higher transcription levels in the double mutants (Figure 6A). In addition, *YUC6* was also significantly increased at the transcriptional level in the leaves of the *SWI3B-amic* mutants compared to that in the wild type. However, there was no significant change in *YUC6* in the *lfr-2* mutant. Furthermore, the double mutants had a similar expression to that of the *lfr-2* mutant (Figure 6A). In addition, the abaxial gene, *FIL*, was decreased at the transcriptional level in the leaves of *lfr-2* and *SWI3B-amic* single and double mutants compared to that in the wild type (Figure 6B). However, there were no significant changes in the *HD-ZIP III* and *KAN* family genes. These results show that LFR and SWI3B play similar roles in the transcriptional regulation of the expression of *IAMT* and *FIL* in Arabidopsis leaves.

### LFR and SWI3B Are Enriched in Chromatins of *FIL* and *IAMT1*

**FIGURE 7:**
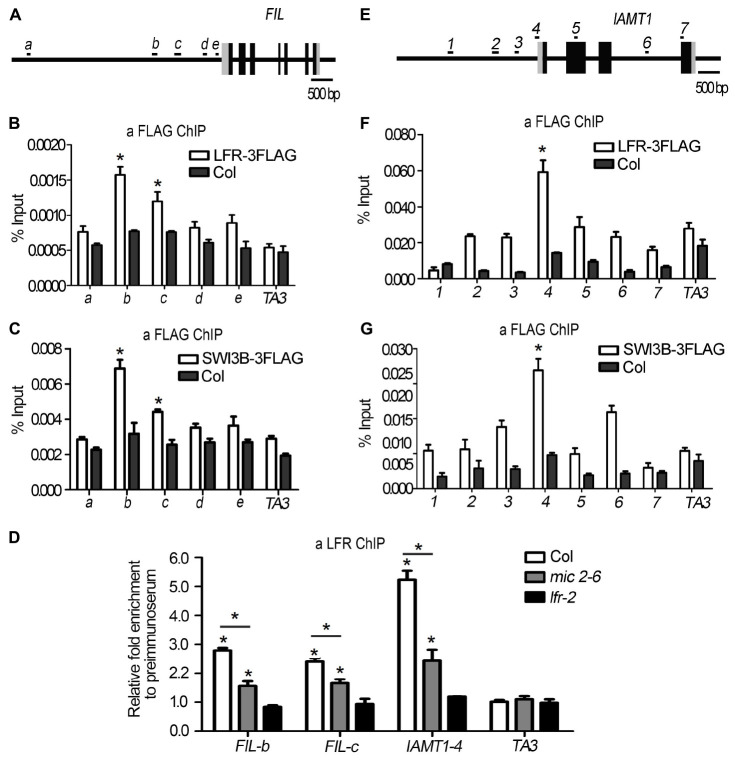
LFR and SWI3B are associated with the chromatin of *FIL* and *IAMT1*. **(A,E)** The diagrams of *FIL* and *IAMT1* gene structures. The black boxes indicate exons, the gray boxes indicate untranslated regions and the long black lines represent the upstream sequence or promoter, introns regions, or 3′-terminal sequence. The lowercase letters **(A)** or the numbers **(E)** and black short lines above the gene structures represent PCR fragments tested in ChIP-qPCR **(B–D,F,G)**. **(B,F)** ChIP-qPCR assay to test the association of LFR-3FLAG with *FIL* **(B)** and IAMT1 **(F)** chromatin using anti-FLAG antibody. **(C,G)** ChIP-qPCR assay to test the association of SWI3B-3FLAG with *FIL* **(C)** and IAMT1 **(G)** locus using anti-FLAG antibody. **(D)** ChIP-qPCR assay to test the association of LFR to *FIL* chromatin using the anti-LFR antibody in *mic2-6*. The bars represent the means of three independent biological repeats and the error bars stand for SE. Significant statistical differences were tested by Student’s *t*-test (*P < 0.05). A retrotransposon locus *TA3* (*At1g37110*) was used as the negative control in ChIP-qPCR **(B–D,F,G)**.

Since we found that the expression of *FIL* was downregulated in both *SWI3B-amic* and *lfr-2* (Figure 6B), we speculated that SWI3B might be a partner of LFR in regulating *FIL* expression. First, we tested whether LFR was tethered to the *FIL* locus by conducting a ChIP-qPCR assay. The upstream *b*-*c* fragments of the *FIL* promoter were reproducibly amplified from the chromatin of *LFR:LFR-FLAG/lfr-2* immunoprecipitated with anti-FLAG; however, no enrichment was detected in Col (Figures 7A,B). To determine whether SWI3B was also tethered to the *FIL* locus, we performed ChIP-qPCR in *35S:SWI3B-FLAG/swi3b-2* transgenic plants, and the significant enrichment of SWI3B-FLAG at *b*-*c* fragments of *FIL* chromatin was reproducibly detected in SWI3B-FLAG fusion protein compared to that in the Col control (Figure 7C). These results suggest that there is an association between LFR and SWI3B and the *FIL* promoter, thereby indicating that *FIL* is the direct target gene of LFR and SWI3B.

To further investigate whether the binding activity of LFR to the *FIL* locus was dependent on SWI3B, we performed a ChIP-qPCR assay in *mic2-6* mutant plants using anti-LFR antibodies. In the absence of functional *SWI3B*, the enrichment of LFR at fragments *b* and *c* of the *FIL* promoter was partly reduced compared to that in the wild type (Figure 7D). To rule out the possibility that the reduction in binding ability might result from low LFR levels in *mic2-6*, we conducted Western blotting and found that the protein level of LFR in *mic2-6* was almost comparable to that in the wild-type control (Figure 5C). These data indicated that LFR and SWI3B co-target the *FIL* locus. Furthermore, the binding of LFR to the *FIL* locus is partly dependent on SWI3B.

In addition, it was reported that the overexpression of *IAMT1* caused upward-curling leaf in *SWI3B-RNAi* plants (Han et al., 2018), but it is not clear that whether SWI3B was associated with the *IAMT1* chromatin. Since *IAMT1* transcript levels were increased in *lfr-2* and *SWI3B-amic* plants (Figure 6A), we tested the association between SWI3B and LFR and the chromatin of *IAMT1*. ChIP-qPCR assay data showed that fragment 4 (−65 to 45) was reproducibly amplified from the chromatin of *LFR:LFR-FLAG/lfr-2* or *35S:SWI3B-FLAG/swi3b-2* transgenic plants immunoprecipitated with anti-FLAG. However, no enrichment was detected in Col (Figures 7E–G). However, we did not detect any enrichment signals of LFR or SWI3B at the *YUC6* locus (Supplementary Figure 5). Moreover, the enrichment of LFR in the chromatin of *IAMT1* was partly reduced in the *mic2-6* mutant compared to that in the wild type (Figure 7D). These results indicate that LFR and SWI3B co-target the *IAMT1* locus *in vivo*. Moreover, the binding activity of LFR to the *IAMT1* locus is partly dependent on SWI3B.

### Increased *FIL* Expression Partially Recovers Upward-Curling Leaf Phenotype of *lfr* Mutant

**FIGURE 8:**
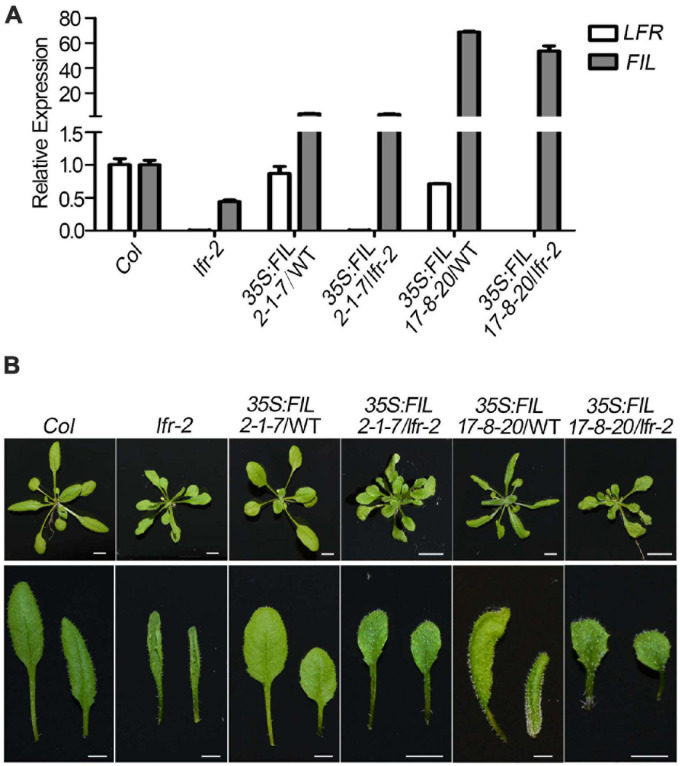
Overexpression of *FIL* partially rescued the upward-curling leaf defect of *lfr*. **(A)** The qRT-PCR data to detect the transcript level of *FIL* in different backgrounds as indicated. The total RNA was isolated from the rosette leaves of 22-day-old Col, *lfr-2*, or the transgenic plants. Transcript levels were normalized to loading control gene *eIF4A1*. One of the three biological replicates with a similar expression pattern was shown. Bars indicate the means ± SE of three technical replicates. **(B)** Genetic interaction between *35S:FIL* and *lfr-2*. The phenotype of whole plants (upper) and the representative rosette leaves (lower) from 22-day-old plants from different genotypes as indicated. Scale bars = 0.5 cm.

To further establish the link between *FIL* expression and the upward-curling leaf phenotype of *lfr*, we conducted the genetic analysis by introducing *35S:FIL* into *lfr-2* heterozygous plant background. The *FIL* expression levels were increased by different degrees in the transgenic lines, *35S:FIL 2-1-7* and *35S:FIL 17-8-20* in both the wild type (WT) and *lfr-2* background (Figure 8A). Though the rosette leaves of *35S:FIL 2-1-7*/WT displayed largely the same morphology as those of the Col, the *35S:FIL 17-8-20*/WT exhibited obviously downward-curling leaf phenotype, which may be resulted from the significant overexpression of *FIL* (Figures 8A,B; Bonaccorso et al., 2012). Intriguingly, the increased expression level of *FIL* can partially recover the upward-curling leaf phenotype of *lfr-2* (Figure 8B). These results indicated that the downregulation of *FIL* may be one of the possible causes of the upward-curling leaf phenotype of *lfr*, which provides genetic evidence for the regulation of *FIL* by LFR. Besides, we also found that the double mutants of *35S:FIL 2-1-7*/*lfr-2* and *35S:FIL 17-8-20*/*lfr-2* had smaller and more leaves than the Col, indicating that there might be some phenotype enhancement in the process of SAM development when overexpressing *FIL* in the *lfr*-2 background.

## Discussion

Our previous study demonstrated that Arabidopsis LFR plays pivotal roles during leaf and flower development (Wang et al., 2009, 2012; Lin et al., 2018). LFR encodes a nuclear protein with ARM-repeat domains (Wang et al., 2009). Through genetic screening, we identified that LFR synergistically interacts with AS2 to repress *BP* expression in the specific processes of leaf development, such as leaf petiole length, the formation of leaf midvein, and elongation of leaflet-like structure at the leaf margin (Lin et al., 2018). However, LFR and AS2 seem to act oppositely in control of the flattened leaf development. To further elucidate the molecular mechanism of LFR in flattened leaf blade development, we isolated the SWI/SNF complex subunit, SWI3B, as another interacting partner of LFR by Y2H screening. This interaction was confirmed by BiFC and co-IP (Figure 1). Y2H and transgenic complementary assays of different truncated LFR proteins showed that the ND domain of LFR was essential for its interaction with SWI3B and was important for its biological function in Arabidopsis (Figures 2, 3). Consistent with a previous report (Han et al., 2018), the knock-down mutant of *SWI3B* by artificial miRNA (*SWI3B-amic*) resulted in an upward-curling leaf phenotype, which was similar to those of the *lfr-1* and *lfr-2* mutations (Figure 4). Different combinations of *lfr-1/lfr-2* and *mic1-2/mic2-6* double mutants also exhibited upward-curling leaves to a little stronger than the single mutants (Figure 5). In addition, LFR and SWI3B co-targeted similar chromatin regions of *FIL* and *IAMT1*, which were differentially expressed in the single and double mutants of *lfr-2*, *SWI3B-amic*, and double mutants (Figures 6, 7). Furthermore, the association between LFR and *FIL* or *IAMT1* was partly dependent on SWI3B (Figure 7D). Interestingly, we notice that the expression of *IAMT1* in *lfr SWI3B-amic* double mutant is higher than either single mutant (Figure 6A), and the curly leaf phenotype of *lfr SWI3B-amic* double mutant seems a little stronger than single mutants (Figures 5D,E), suggesting that besides the functional LFR-SWI3B complex, LFR might also regulate *IAMT1* expression independently of SWI3B. Taken together, our results demonstrate that LFR and SWI3B physically interact to directly regulate the expression of *FIL* and *IAMT1*. This provides an epigenetic mechanism underlying the development of flattened leaf lamina in Arabidopsis. The main difference between the current findings and our previous report (Lin et al., 2018) is that: our current findings revealed that the flattened leaf blade is regulated by LFR-SWI3B; our previous report showed that LFR interacts with AS2 to control the leaf petiole length, the formation of leaf midvein, and the elongation of leaflet-like structure at the leaf margin (Lin et al., 2018). Altogether, these explained the molecular mechanism underlying different aspects of Arabidopsis leaf development.

It was reported that RNAi mutants of *SWI3B* resulted in an upward-curling leaf phenotype resulting from decreased nucleosome occupation and increased transcript level of *IAMT1* (Han et al., 2018). However, whether *IAMT1* was the direct target of SWI3B and other targets of SWI3B and its interacting partners in leaf development remains largely unclear. In this study, we further identified LFR as the interacting partner of SWI3B in flattened leaf development and showed that *IAMT1* was a target of both LFR and SWI3B. In addition, we found that both proteins co-targeted *FIL*, which was a critical transcription factor involved in abaxial cell fate determination. Our previous work showed that LFR interacts with AS2 (Lin et al., 2018) and it was also demonstrated that the AS1-AS2 complex is functionally associated with the histone deacetylase HDA6 to regulate leaf development (Luo et al., 2012). Recently, it was reported that SWI3B interacts with HDA6 to maintain transposon silencing in Arabidopsis (Yang et al., 2020). And we found in this study that LFR interact with SWI3B and *SWI3B-amic* displayed a similar leaf margin phenotype (a sawtooth appearance) to *lfr* (Figure 4). All these results mutually supported that the ARM repeat domain-containing protein LFR might integrate the actions of transcription factors and epigenetic regulators into a concerted transcriptional complex to regulate the expression of some common target(s), such as *BP*. Furthermore, it was reported that the MONOPTEROS (MP/ARF5) transcription factor recruited the SWI/SNF chromatin remodelers, BRAHMA (BRM) and SPLAYED (SYD), to increase DNA accessibility of *FIL* for the induction of flower primordium initiation (Wu et al., 2015). In different tissues, both LFR-SWI3B and MP-BRM/SYD bind to similar regions of the *FIL* promoter (b and c loci). Therefore, the SWI/SNF chromatin remodeling complex members may play similar roles in regulating *FIL* expression in both leaf and flower (lateral organ) development. Therefore, it was interesting to test whether MP or other transcription factors recruited the LFR-SWI3B complex to the target genes.

Although *BRM* and *SYD* play similar positive regulatory roles on *FIL* in flower primordium initiation to that of LFR-SWI3B in leaf development, the *brm* and *syd* mutants displayed a downward-curling leaf phenotype (Sarnowski et al., 2005; Sacharowski et al., 2015), which was opposite to the phenotype of *LFR* loss-of-function and *SWI3B* knock-down mutants (Figure 4). The possible explanations are as follows: (1) the detailed tissue- or cell type-specific expression of *FIL* and other possible target genes may be different in different genotype backgrounds. (2) previous studies indicate that, in addition to similar functions, SWI/SNF subunits display distinct functions, such as those of SWI/SNF ATPase (e.g., BRM and SYD), SWI3 proteins (SWI3A, SWI3B, SWI3C, and SWI3D), and SWP73 (SWP73A and SWP73B) (Sarnowski et al., 2005; Bezhani et al., 2007; Sacharowski et al., 2015). In this study, we detected the physical interaction between LFR and SWI3B and possibly SWI3A. However, we did not detect any physical interaction between LFR and SWI3C and SWI3D (Figure 4 and Supplementary Figure 1). These results suggest that LFR, SWI3B, and/or SWI3A may act in the same SWI/SNF chromatin remodeling complex in Arabidopsis leaf development. BRM is an ATPase subunit of the SWI/SNF complex. It physically interacts with SWI3C and SWP73B. A loss-of-function mutant exhibits a downward-curling leaf phenotype similar to that of *brm* (Hurtado et al., 2006; Sacharowski et al., 2015). Therefore, BRM, SWP73B, and SWI3C may be present in the same SWI/SNF complex in maintaining a flattened development process; LFR and SWI3B/3A may be present in another type of SWI/SNF complex, including another ATPase. (3) It is also possible that LFR-SWI3B may have functions independent of the SWISNF complex. It is interesting to uncover the mechanisms underlying the differences in leaf phenotypes in these mutants. Whether LFR is a constant component of the SWI/SNF complex and the composition of different SWI/SNF complex in different tissues and developmental stages still need further investigation, which would shed light on the biochemical composition of SWI/SNF complex and the epigenetic control of plant development.

## Conclusion

The results of our study indicate that LFR physically interacts with SWI3B, a core component of the SWI/SNF chromatin remodeling complex, and with the ND domain of LFR, which is responsible for the interaction between LFR and SWI3B. This interaction is crucial for LFR functions in Arabidopsis. Results of the genetic analysis further reveal that *lfr* and *SWI3B-amic* single and double mutants have upward-curling leaf phenotypes. This phenotype is similar to those with altered *FIL* and *IAMT1* expression. Moreover, the results of further experiments show that LFR binds to the chromatin of *FIL* and *IAMT1* and are partly dependent on SWI3B. And overexpression of *FIL* partly recovers the curly leaf defect of *lfr*. Taken together, LFR interacts with SWI3B to regulate *FIL* and *IAMT1* expression and maintains the normal leaf blade development process.

## Data Availability Statement

The original contributions presented in the study are included in the article/Supplementary Material, further inquiries can be directed to the corresponding author/s.

## Author Contributions

SC and HZ planned and conceptualized the study and designed the experiments. XWL performed the BiFC, western blot assay, co-IP, ChIP-qPCR, partial phenotypic analysis, and qRT-PCR. CY did the Y2H, phenotypic analysis, and RT-PCR. BZ and SY performed the partial phenotypic analysis and transgenic plant screening. TY conducted the *35S:LFR-3FLAG/lfr-1* plasmid construction and partial phenotypic analysis. XRL performed plasmid construction. XWL, CY, HZ, and SC wrote the manuscript with input from co-authors. All authors contributed to the article and approved the submitted version.

## Conflict of Interest

The authors declare that the research was conducted in the absence of any commercial or financial relationships that could be construed as a potential conflict of interest.

## Publisher’s Note

All claims expressed in this article are solely those of the authors and do not necessarily represent those of their affiliated organizations, or those of the publisher, the editors and the reviewers. Any product that may be evaluated in this article, or claim that may be made by its manufacturer, is not guaranteed or endorsed by the publisher.

## Footnotes

1
http://wmd3.weigelworld.org/cgi-bin/webapp.cgi

2
http://www.clontech.com/takara

## Supplementary Material

Supplementary Figure 1 Transgenic rescue lines of *35S:LFR-3FLAG/lfr-1*.

Supplementary Figure 2 Transgenic rescue lines of *35S:SWI3B-3FLAG/3b-2*.

Supplementary Figure 3 The interaction analysis between LFR and SWI3A, SWI3C, and SWI3D in yeast.

Supplementary Figure 4 Self-activation analysis truncated LFR and SWI3B in yeast.

Supplementary Figure 5 LFR and SWI3B did not associated with the chromatin of *YUC6*.

Supplementary Table 1 Primers used in this paper.

Supplementary Table 2 Potential LFR-interacting proteins identified by the Y2H screening.
