# Corrigendum: LFR Physically and Genetically Interacts With SWI/SNF Component SWI3B to Regulate Leaf Blade Development in Arabidopsis

**DOI:** 10.3389/fpls.2022.901613

**Published:** 2022-04-13

**Authors:** Xiaowei Lin, Can Yuan, Bonan Zhu, Tingting Yuan, Xiaorong Li, Shan Yuan, Sujuan Cui, Hongtao Zhao

**Affiliations:** ^1^Hebei Key Laboratory of Molecular and Cellular Biology, Key Laboratory of Molecular and Cellular Biology of Ministry of Education, Hebei Collaboration Innovation Center for Cell Signaling and Environmental Adaptation, College of Life Sciences, Hebei Normal University, Shijiazhuang, China; ^2^School of Traditional Chinese Medicine, Tianjin University of Traditional Chinese Medicine, Tianjin, China

**Keywords:** LFR, SWI3B, *FIL*, *IAMT1*, SWI/SNF, leaf, Arabidopsis

In the original article, there was a mistake in Figure 7A as published. One extra exon was drawn in the gene model of *FIL* gene in Figure 7A. The corrected Figure 7A appears below.

**Figure 7:**
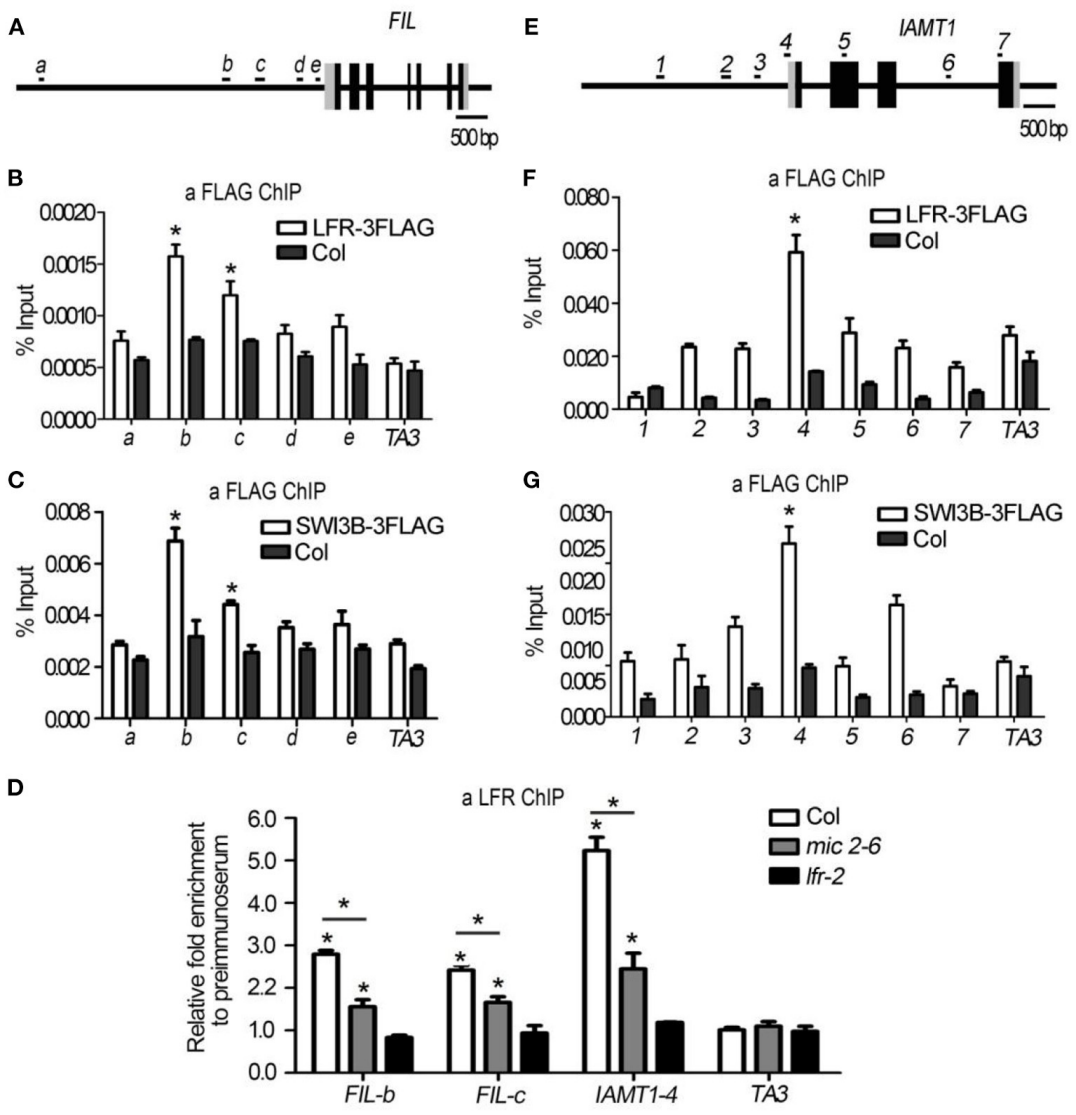
LFR and SWI3B are associated with the chromatin of *FIL* and *IAMT1*. **(A,E)** The diagrams of *FIL* and *IAMT1* gene structures. The black boxes indicate exons, the gray boxes indicate untranslated regions and the long black lines represent the upstream sequence or promoter, introns regions, or 3′-terminal sequence. The lowercase letters **(A)** or the numbers **(E)** and black short lines above the gene structures represent PCR fragments tested in ChIP-qPCR **(B–D,F,G)**. **(B,F)** ChIP-qPCR assay to test the association of LFR-3FLAG with *FIL* **(B)** and *IAMT1* **(F)** chromatin using anti-FLAG antibody. **(C,G)** ChIP-qPCR assay to test the association of SWI3B-3FLAG with *FIL* **(C)** and *IAMT1* **(G)** locus using anti-FLAG antibody. **(D)** ChIP-qPCR assay to test the association of LFR to *FIL* chromatin using the anti-LFR antibody in *mic2-6*. The bars represent the means of three independent biological repeats and the error bars stand for SE. Significant statistical differences were tested by Student's *t*-test (*P < 0.05). A retrotransposon locus *TA3* (*At1g37110*) was used as the negative control in ChIP-qPCR **(B–D,F,G)**.

The authors apologize for this error and state that this does not change the scientific conclusions of the article in any way. The original article has been updated.

## Publisher's Note

All claims expressed in this article are solely those of the authors and do not necessarily represent those of their affiliated organizations, or those of the publisher, the editors and the reviewers. Any product that may be evaluated in this article, or claim that may be made by its manufacturer, is not guaranteed or endorsed by the publisher.

