# Supplementary material for: LFR Physically and Genetically Interacts With SWI/SNF Component SWI3B to Regulate Leaf Blade Development in Arabidopsis
Source: Front Plant Sci. 2021 Aug 11;12:717649. doi: 10.3389/fpls.2021.717649 (PMC8385146; doi:10.3389/fpls.2021.717649)
Supplement: Supplementary Figure 1 — Transgenic rescue lines of 35S:LFR-3FLAG/lfr-1. [file Data_Sheet_1.zip › Supplementary Figures 1-5.DOCX]

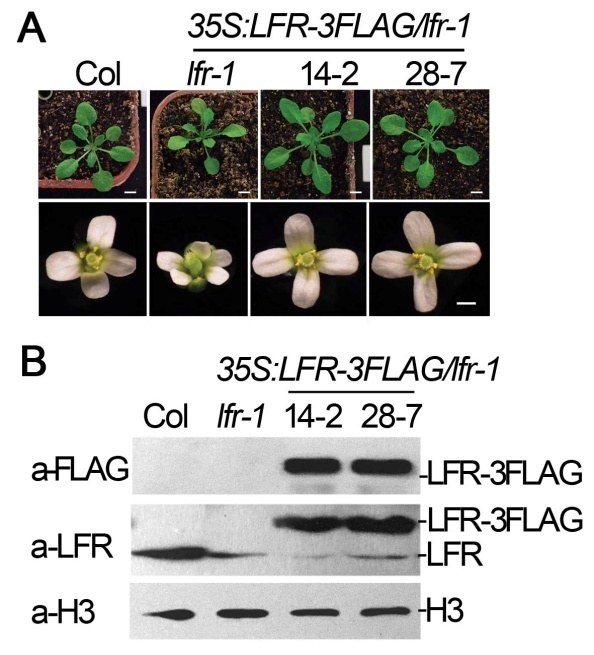


**Supplementary Figure 1.** Transgenic rescue lines of *35S:LFR-3FLAG/lfr-1*.

**(A)** The overall morphology of 18-day-old plants (upper panel) and stage-13 flower (bottom panel) of wild type (Col), *lfr-1*, *35S:LFR-FLAG/lfr-1* under long-day condition. Scale bars in 18-day-old plant and flower pictures = 0.5 cm and 1 mm, respectively.

**(B)** Western blot assays were performed to analyze the protein level of LFR-FLAG and native and transgenic LFR by anti-FLAG (a-FLAG) and anti-LFR (a-LFR), respectively. Anti-Histone 3 (a-H3) was used as a loading control.


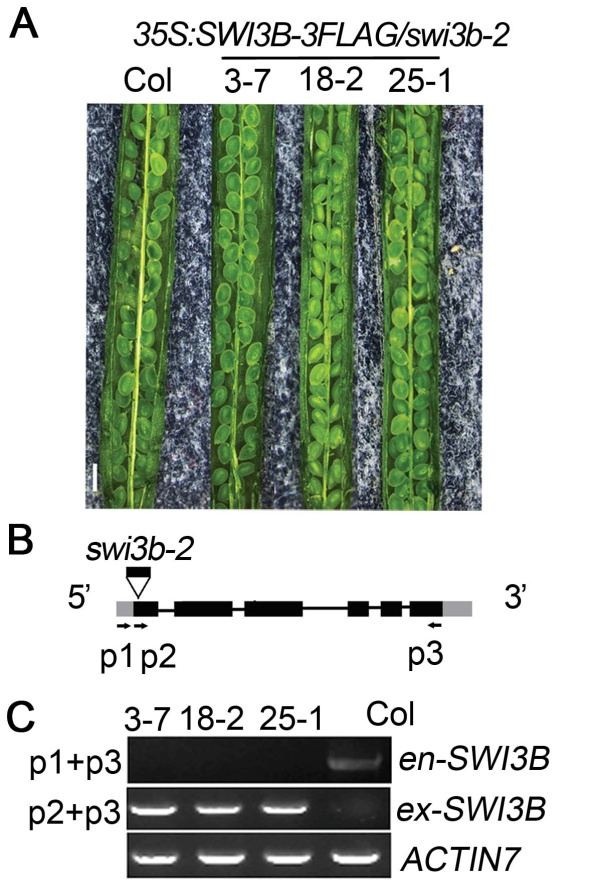


**Supplementary Figure 2.** Transgenic rescue lines of *35S:SWI3B-3FLAG/3b-2*.

**(A)** The siliques of Col and three 3*5S::SWI3B-3FLAG* transgenic rescue lines, 3-7, 18-2 and 25-1, at 7 days after pollination. Bar=100 μm.

**(B)** Schematic of *SWI3B* gene structure showing exons (black thick bars), introns (black thin lines), 5′ and 3′ untranslated regions (gray bars), and T-DNA insertion (triangle) in the *atswi3b-2* mutants. The p1-p3 represent the forward and reverse primers used for RT-PCR analysis.

**(C)** RT-PCR analysis of endogenous *SWI3B* (en-*SWI3B*) using p1 and p3 primers or exogenous *SWI3B-FLAG* (e*x-SWI3B*) transcripts with p2 and p3 primers. *ACTIN7* was used as the loading control.


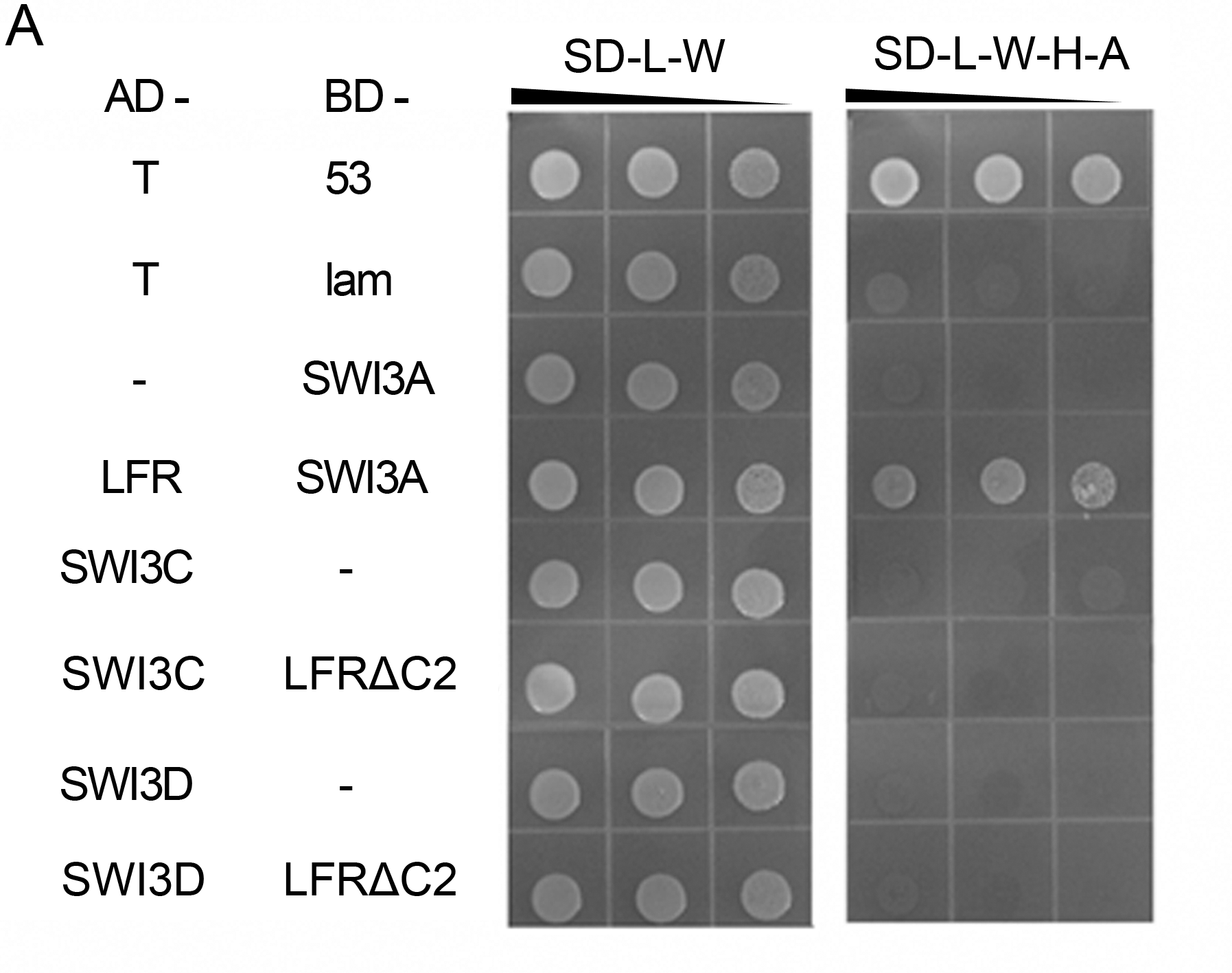


**Supplementary Figure 3.** The interaction analysis between LFR and SWI3A, SWI3C, and SWI3D in yeast.

The plasmid pGBKT7 or plasmid pGADT7 with or without indicated proteins were co-transformed into AH109 on SD-L-W or SD-L-W-H-A after gradient dilution (10^-1^, 10^-2^, 10^-3^) as indicated by black triangles.


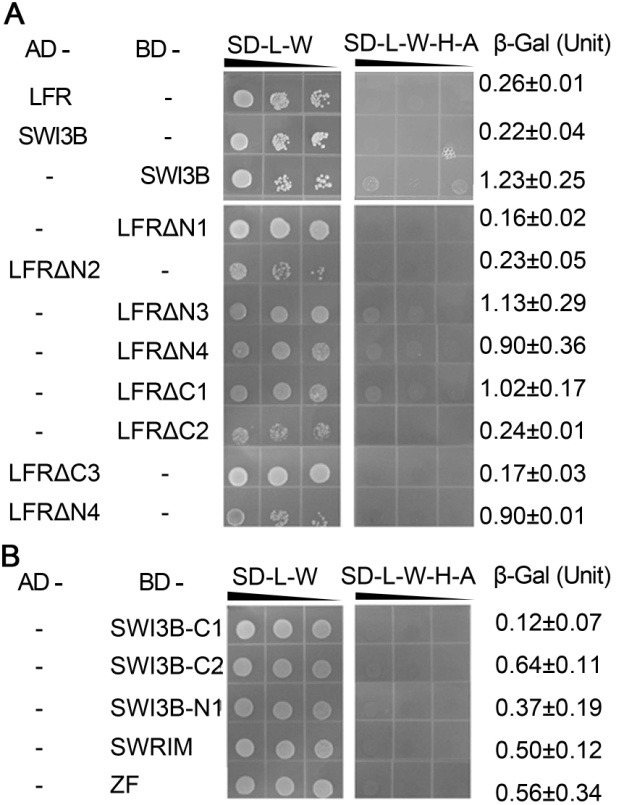


**Supplementary Figure 4.** Self-activation analysis truncated LFR and SWI3B in yeast.

**(A-B)** The self-activation assay truncated LFR (A) and truncated AtSWI3B (B) in yeast. The plasmid pGBKT7 or plasmid pGADT7 with or without indicated proteins were co-transformed into AH109 on SD-L-W or SD-L-W-H-A after gradient dilution (10^-1^, 10^-2^, 10^-3^) as indicated by black triangles. Numbers on the right represent mean ± standard error of three biological replicates of the β-Gal activity.


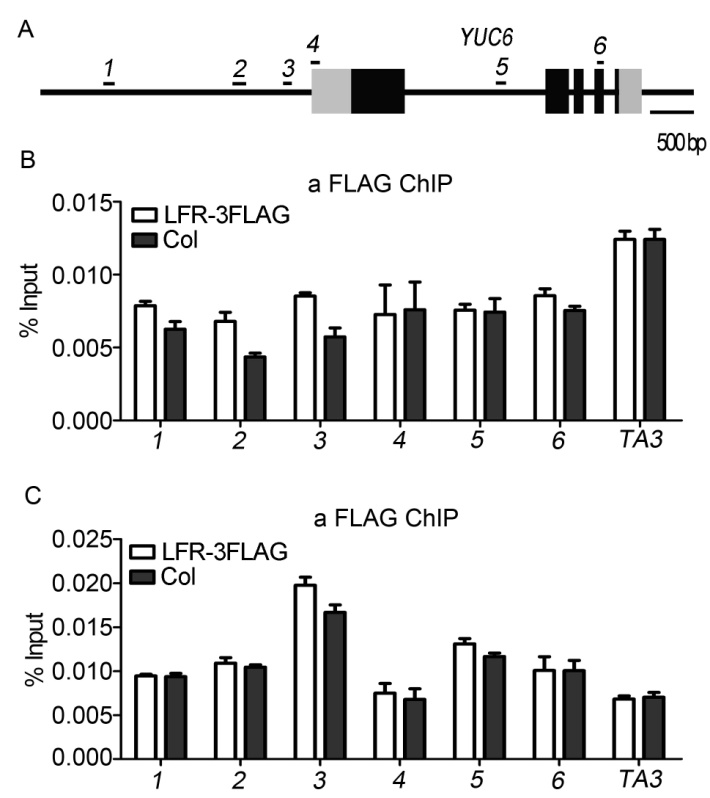


**Supplementary Figure 5.** LFR and SWI3B did not associated with the chromatin of *YUC6*.

**(A)** The diagrams of *YUC6* gene structures. The black boxes indicate exons, the gray boxes indicate untranslated regions, and the long black lines represent the upstream sequence or promoter, introns regions or 3’-terminal sequence. The numbers and black short lines above the gene structures represent PCR fragments tested in ChIP-qPCR.

**(B-C)** ChIP-qPCR assay to test the association of LFR-3FLAG (B) and SWI3B-3FLAG (E) with *YUC6* chromatin using anti-FLAG antibody. The bars represent means of three independent biological repeats and the error bars stand for SE. Significant statistical differences were tested by Student’s *t*-test (**P* < 0.05). A retrotransposon locus *TA3* (*At1g37110*) was used as the negative control.
