# Supplementary material for: LFR Physically and Genetically Interacts With SWI/SNF Component SWI3B to Regulate Leaf Blade Development in Arabidopsis
Source: Front Plant Sci. 2021 Aug 11;12:717649. doi: 10.3389/fpls.2021.717649 (PMC8385146; doi:10.3389/fpls.2021.717649)
Supplement: Supplementary Figure 1 — Transgenic rescue lines of 35S:LFR-3FLAG/lfr-1. [file Data_Sheet_1.zip › Supplementary Table 1.DOCX]

**Table S1. List of primers used in this paper**

| Primers | Sequence(5‘-3’) | | | | Description |  |
| --- | --- | --- | --- | --- | --- | --- |
| pGBKT7 GBD-LFR-FP | CGGAATTCATGCAGAAACGGGAGCTTG | | | | construct Y2H vectors |  |
| pGBKT7 GBD-LFR-RP | ACGCGTCGACTTACATGCCCCAGATTCCTCTAG | | | |  |  |
| pGBKT7 GBD-LFR△C1-FP | CGGAATTCATGCAGAAACGGGAGCTTG | | | |  |  |
| pGBKT7 GBD-LFR△C1-RP | ACGCGTCGACTCAGGCTAGAGCATCATACTCATTGC | | | |  |  |
| pGBKT7 GBD-LFR△C2-FP | CGGAATTCATGCAGAAACGGGAGCTTG | | | |  |  |
| pGBKT7 GBD-LFR△C2-RP | ACGCGTCGACTCAAGCAGCACAATTCCAAGC | | | |  |  |
| pGBKT7 GBD-LFR△C3-FP | CGGAATTCATGCAGAAACGGGAGCTTG | | | |  |  |
| pGBKT7 GBD-LFR△C3-RP | ACGCGTCGACTCAGAGATTGTAGAGTGCTCCAAC | | | |  |  |
| pGBKT7 GBD-LFR△C4-FP | CGGAATTCATGCAGAAACGGGAGCTTG | | | |  |  |
| pGBKT7 GBD-LFR△C4-RP | ACGCGTCGACTCATTGAGGCTCCGAGACAAGG | | | |  |  |
| pGBKT7 GBD-LFR△N1-FP | CCGGAATTCATGAATTCCTTCGTCGAGCAG | | | |  |  |
| pGBKT7 GBD-LFR△N1-RP | ACGCGTCGACTTACATGCCCCAGATTCCTCTAG | | | |  |  |
| pGBKT7 GBD-LFR△N2-FP | CCGGAATTCATGCCCTTCGGTAGTACAAGTGC | | | |  |  |
| pGBKT7 GBD-LFR△N2-RP | ACGCGTCGACTTACATGCCCCAGATTCCTCTAG | | | |  |  |
| pGBKT7 GBD-LFR△N3-FP | CCGGAATTCATGAATTCCTTCGTCGAGCAG | | | |  |  |
| pGBKT7 GBD-LFR△N3-RP | ACGCGTCGACTCAGAGATTGTAGAGTGCTCCAAC | | | |  |  |
| pGBKT7 GBD-LFR△N4-FP | CCGGAATTCATGAATTCCTTCGTCGAGCAG | | | |  |  |
| pGBKT7 GBD-LFR△N4-RP | ACGCGTCGACTCATTGAGGCTCCGAGACAAGG | | | |  |  |
| pGADT7 GAD-LFR-FP | CGGAATTCATGCAGAAACGGGAGCTTG | | | |  |  |
| pGADT7 GAD-LFR-RP | CCGCTCGAGTTACATGCCCCAGATTCCTCTAG | | | |  |  |
| pGADT7 GAD-LFR△C3-FP | CGGAATTCATGCAGAAACGGGAGCTTG | | | |  |  |
| pGADT7 GAD-LFR△C3-RP | CCGCTCGAGTCAGAGATTGTAGAGTGCTCCAAC | | | |  |  |
| pGADT7 GAD-LFR△C4-FP | CGGAATTCATGCAGAAACGGGAGCTTG | | | |  |  |
| pGADT7 GAD-LFR△C4-RP | CCGCTCGAGTCATTGAGGCTCCGAGACAAGG | | | |  |  |
| pGADT7 GAD-LFR△N2-FP | CCGGAATTCATGCCCTTCGGTAGTACAAGTGC | | | |  |  |
| pGADT7 GAD-LFR△N2-RP | CCGCTCGAGTTACATGCCCCAGATTCCTCTAG | | | |  |  |
| pGBKT7 GBD-SWI3B-FP | CGGAATTCATGGCCATGAAAGCTCC | | | |  |  |
| pGBKT7 GBD-SWI3B-RP | ACGCGTCGACCTAACACTCTATTCTATCTTCAG | | | |  |  |
| pGBKT7 GBD-SWI3B-C1-FP | CGGAATTCATGGCCATGAAAGCTCC | | | |  |  |
| pGBKT7 GBD-SWI3B-C1-RP | CGGGATCCCTAAGCTGCTGCTTCTGC | | | |  |  |
| pGBKT7 GBD-SWI3B-C2-FP | CGGAATTCATGGCCATGAAAGCTCC | | | |  |  |
| pGBKT7 GBD-SWI3B-C2-RP | CGGGATCCCTAAATCTCAACTCGTTTAAAC | | | |  |  |
| pGBKT7 GBD-SWI3B-N1-FP | CGGAATTCAGTGAGGAGTCAAAGCCAGAG | | | |  |  |
| pGBKT7 GBD-SWI3B-N1-RP | ACGCGTCGACCTAACACTCTATTCTATCTTCAG | | | |  |  |
| pGBKT7 GBD-SWIRM-FP | CGGAATTCATGGCCATGAAAGCTCC | | | |  |  |
| pGBKT7 GBD-SWIRM-RP | CGGGATCCCTAGGCGGAGCTATTATAGTTG | | | |  |  |
| pGBKT7 GBD-ZF-FP | CGGAATTCATCAACTATAATAGCTCCGCC | | | |  |  |
| pGBKT7 GBD-ZF-RP | CGGGATCCCTAAATCTCAACTCGTTTAAACTC | | | |  |  |
| pGADT7 GAD-SWI3B-FP | CGGAATTCATGGCCATGAAAGCTCC | | | |  |  |
| pGADT7 GAD-SWI3B-RP | CGGGATCCCTAACACTCTATTCTATCTTCAG | | | |  |  |
| pCAMBIA1300-35S:LFR-3FLAG-FP | | | TCCCCCGGGATGCAGAAACGGGAGCTTG | | Construct vectors for transgenic lines |  |
| pCAMBIA1300-35S:LFR-3FLAG-RP | | | TCCCCCGGGCATGCCCCAGATTCCTCTAG | |  |  |
| pCAMBIA1300-35S:LFR△N1-3FLAG-FP | | | GGACTAGTATGAATTCCTTCGTCGAGCAG | |  |  |
| pCAMBIA1300-35S:LFR△N1-3FLAG-RP | | | GGACTAGTCATGCCCCAGATTCCTCTAG | |  |  |
| pCAMBIA1300-35S:LFR△N2-3FLAG -FP | | | TCCCCCGGGATGCCCTTCGGTAGTACAAGTGC | |  |  |
| pCAMBIA1300-35S:LFR△N2-3FLAG-RP | | | TCCCCCGGGCATGCCCCAGATTCCTCTAG | |  |  |
| pCAMBIA1300-35S:LFR△C1-3FLAG-FP | | | GGATCCATGCAGAAACGGGAGCTTG | |  |  |
| pCAMBIA1300-35S:LFR△C1-3FLAG-RP | | | GGATCCGGCTAGAGCATCATACTCATTGC | |  |  |
| pCAMBIA1300-35S:LFR△C3-3FLAG-FP | | | TCCCCCGGGATGCAGAAACGGGAGCTTG | |  |  |
| pCAMBIA1300-35S:LFR△C3-3FLAG-RP | | | TCCCCCGGGGAGATTGTAGAGTGCTCCAAC | |  |  |
| pCAMBIA1300-35S:LFR△C4-3FLAG-FP | | | TCCCCCGGGATGCAGAAACGGGAGCTTG | |  |  |
| pCAMBIA1300-35S:LFR△C4-3FLAG-RP | | | TCCCCCGGGTTGAGGCTCCGAGACAAGG | |  |  |
| pCAMBIA1300-35S:LFR-FLAG-FP | | | CCCCGGGATGCAGAAACGGGAGCTTG | |  |  |
| pCAMBIA1300-35S:LFR-FLAG-RP | | | CCCCGGGCATGCCCCAGATTCCTCTAG | |  |  |
| pCAMBIA1300-35S:SWI3B-3FLAG-FP | | | GGATCCATGGCCATGAAAGCTCC | |  |  |
| pCAMBIA1300-35S:SWI3B-3FLAG-RP | | | GGATCCACACTCTATTCTATCTTCAG | |  |  |
| pAVA321-35S:SWI3B-CFP-FP | | | GACTAGTATGGCCATGAAAGCTCC | |  |  |
| pAVA321-35S: SWI3B-CFP-RP | | | CATGCCATGGCACACTCTATTCTATCTTCAG | |  |  |
| p1300-FIL-FP | | | GCTCTAGAATGTCTATGTCGTCTATGTCCTCC | |  |  |
| p1300-FIL-RP | | | CGGGATCCTTAATAAGGAGTCACACCAACGTTAG | |  |  |
| ⅠmiR-s | | gaTTACTACGAACATAGCACCCCtctctcttttgtattcc | | | pRS300 mic1 |  |
| Ⅱ miR-a | | gaGGGGTGCTATGTTCGTAGTAAtcaaagagaatcaatga | | |  |  |
| Ⅲ miR*s | | gaGGAGTGCTATGTTGGTAGTATtcacaggtcgtgatatg | | |  |  |
| Ⅳ miR*a | | gaATACTACCAACATAGCACTCCtctacatatatattcct | | |  |  |
| ⅠmiR-s | | gaTTTCTACCAATAACATGCGACtctctcttttgtattcc | | | pRS300 mic2 |  |
| Ⅱ miR-a | | gaGTCGCATGTTATTGGTAGAAAtcaaagagaatcaatga | | |  |  |
| Ⅲ miR*s | | gaGTAGCATGTTATTCGTAGAATtcacaggtcgtgatatg | | |  |  |
| Ⅳ miR*a | | gaATTCTACGAATAACATGCTACtctacatatatattcct | | |  |  |
| GW-SWI3B-FP | | | CACCATGGCCATGAAAGCTCCC | | Constructs for BIFC |  |
| GW-SWI3B-RP | | | CTAACACTCTATTCTATCTTCAGTTTTCCG | |  |  |
| qSWI3C-FP | | | CGGCGGCGAGGAAAC | | qRT-PCR |  |
| qSWI3C-RP | | | CGGCATTTTCAACGTCATC | |  |  |
| qSWI3D-FP | | | CTCTGGAGATTCTCCGGC | |  |  |
| qSWI3D-RP | | | CTCTTCGACGGCGCAGAA | |  |  |
| qSWI3A-FP | | | GCGGAGTTCTTCACTG | |  |  |
| qSWI3A-RP | | | GTGAAGGTGAGTCTCCG | |  |  |
| qSWI3B-FP | | | CCTAGCTACTCCAGTTGG | |  |  |
| qSWI3B-RP | | | TAGGGTTTTTGGATGAAG | |  |  |
| qLFR-FP | | | CTGTTGGAGCACTCTACAATCTCG | |  |  |
| qLFR-RP | | | GGATGCGGAGTCTTTATCACTTTC | |  |  |
| qIAMT1-FP | | | CCAGCGAGAACCATTGACTTTTTCC | |  |  |
| qIAMT1-RP | | | TCTCTCCGGCACCGTGTATGAAAAC | |  |  |
| qFIL-FP | | | TATGTCGTCTATGTCCTCCCC | |  |  |
| qFIL-RP | | | GAACATTAACCGCAAGGATGG | |  |  |
| qYUC6-FP | | | GATCTCTGCAACTTCGGTGCTCAG | |  |  |
| qYUC6-RP | | | GATGGGCAGCCATTTCAGTAAGAAC | |  |  |
| qIAA17-FP | | | CGTCGTGACTTTTGATTCCAAGGAG | |  |  |
| qIAA17-RP | | | CCGCTTGATTTTTGGCAGGAAAC | |  |  |
| qIAA3-FP | | | GAGGCTGGGATTACCGGGAACA | |  |  |
| qIAA3-RP | | | CATCCAACAATCTGAGCCTTTCGAG | |  |  |
| ACTIN7-FP | | | AGGCACCTCTTAACCCTAAAGC | |  |  |
| *ACTIN7*-RP | | | GGACAACGGAATCTCTCAGC | |  |  |
| eIF4A1-FP | | | AAACTCAATGAAGTACTTGAGGGACA | |  |  |
| eIF4A1-RP | | | CAAGATTCTCACTGGCTTGCTC | |  |  |
| lfr-2-FP | | | | GAGAAGGGCATCAAGCAAGC | | Identification of mutants in DNA level |
| lfr-2-RP | | | | GGTTTGGCGATTTCCCTCTGC | |  |
| P8409 | | | | CCATATTGACCATCATACTCATTGC | |  |
| swi3b-2-FP | | | | GCGAAGTTGCGTTAGTTAAACAGAG | |  |
| Swi3b-2-RP | | | | CTTATCACAGGCGAAACAAGCTATG | |  |
| LB (GABI-LEFT) | | | | TCTCCATATTGACCATCATACTCATTGC | |  |
| P1 | | | GCGAAGTTGCGTTAGTTAAACAGAG | | Identificati endogenous or exogenous of SWI3B gene in DNA level | |
| P2 | | | GGATCCATGGCCATGAAAGCTCC | |  |  |
| P3 | | | GGATCCACACTCTATTCTATCTTCAG | |  |  |
| ACTIN7-FP | | | AGGCACCTCTTAACCCTAAAGC | |  |  |
| ACTIN7-RP | | | GGACAACGGAATCTCTCAGC | |  |  |
| LFR-f | | | | ATGCAGAAACGGGAGCTTG | | The primers for LFR in RT-PCR analysis |
| LFR-r | | | | TTACATGCCCCAGATTCCTCTAG | |  |
| C1-f | | | | ATGCAGAAACGGGAGCTTG | |  |
| C3-f | | | | ATGCAGAAACGGGAGCTTG | |  |
| C4-f | | | | ATGCAGAAACGGGAGCTTG | |  |
| N1-f | | | | ATGAATTCCTTCGTCGAGCAG | |  |
| N2-f | | | | ATGCCCTTCGGTAGTACAAGTGC | |  |
| FLAG-r | | | | TCGTCATCGTCTTTGTAGTCCAT | |  |
| FILChIP-a-FP | | | | GATAGAAAATAGAACTGGAGAAACCCT | | The ChIP primers for *FIL* |
| FILChIP-a-RP | | | | GTGAATAAAGCATTTACTTATTCTGTGC | |  |
| FILChIP/FAIRE-b-FP | | | | tcttcctccattgggaactg | |  |
| FILChIP/FAIRE-b-RP | | | | ggttgcacgtgatgacacat | |  |
| FILChIP-c-FP | | | | GAAAAAGGTCCATCTTTAAGCG | |  |
| FILChIP-c-RP | | | | Agaaattcggttgaccatgc | |  |
| FILChIP-d-FP | | | | Ttgtcttattaaacaccggcata | |  |
| FILChIP-d-RP | | | | Agcggtagtgaaccagcaac | |  |
| FILChIP-e-FP | | | | GACCTAGAGGCTACAATTAGAGCAT | |  |
| FILChIP-e-RP | | | | GGTGAAAGGAGTGAGAAGGGAAA | |  |
| FILChIP-f-FP | | | | ATGTCTATGTCGTCTATGTCCTCC | |  |
| FILChIP-f-RP | | | | GGTCCGGTGAACAAACAGC | |  |
| FILChIP-g-FP | | | | TATGTCCTCCCAGCTTCTAACC | |  |
| FILChIP-g-RP | | | | GAAGTAAGAGTGAGGACCGAGCT | |  |
| FILChIP-h-FP | | | | attgccagCTCCAGAGAAAA | |  |
| FILChIP-h-RP | | | | TAAGAGACTCACTTGATGAATCGG | |  |
| FILChIP-i-FP | | | | CGTTGGTGTGACTCCTTATTAAaga | |  |
| FILChIP-i-RP | | | | GACATGATAAACCCTAAGAAATTGGA | |  |
| TA3-FP | | | | CTGCGTGGAAGTCTGTCAAA | |  |
| TA3-RP | | | | CTATGCCACAGGGCAGTTTT | |  |
| ChIP-IAMT1-1-FP | | | | Gtagattgtagattacgagcataaagaagtgtg | | The ChIP primers for *IAMT1* |
| ChIP-IAMT1-1-RP | | | | Cttcagatttccttaatttgcttgttcttacac | |  |
| ChIP-IAMT1-2-FP | | | | Gggtggtagcctatggaaacacaat | |  |
| ChIP-IAMT1-2-RP | | | | Gtcttaagagtaagaacctattggttggc | |  |
| ChIP-IAMT1-3-FP | | | | Gtgctgctttatagaaaagtaaagttaacaca | |  |
| ChIP-IAMT1-3-RP | | | | Tcttaaaatgacatttctgttaagtattatcgc | |  |
| ChIP-IAMT1-4-FP | | | | Gtacgcaatggcacatttaataatatgtttcc | |  |
| ChIP-IAMT1-4-RP | | | | Gctcgaggagacgacacatatatatagga | |  |
| ChIP-IAMT1-5-FP | | | | CTCTAAGAGGTTTGATGCTGCCG | |  |
| ChIP-IAMT1-5-RP | | | | GACAAGTGGTGGAAGAAGCTGG | |  |
| ChIP-IAMT1-6-FP | | | | Gccggccctgagtaacaatatata | |  |
| ChIP-IAMT1-6-RP | | | | gtcgttaaacttcatttcaagaaacttgtgga | |  |
| ChIP-IAMT1-7-FP | | | | GttttatagGGTTTGGTAGCAGCAGAG | |  |
| ChIP-IAMT1-7-RP | | | | CAATTGCAAATGAGCCGTTGGC | |  |
| ChIP-YUC6-1-FP | | | | gcatgcagtaattctatttcggttgtactg | | The ChIP primers for *YUC6* |
| ChIP-YUC6-1-RP | | | | accaactagaatttgttactagctaaagctagtc | |  |
| ChIP-YUC6-2-FP | | | | gtgtaaaaattacacaagcctaatcgaaacc | |  |
| ChIP-YUC6-2-RP | | | | gagaaatgggtgcaattagagtaagacatc | |  |
| ChIP-YUC6-3-FP | | | | gcagccattggttgatcataagaaaac | |  |
| ChIP-YUC6-3-RP | | | | tggtgttccactatacttttgactctttct | |  |
| ChIP-YUC6-4-FP | | | | aaaactaacaatctctctctctctttcc | |  |
| ChIP-YUC6-4-RP | | | | TGTGTTTGTGTTTTGATGAGAGTGT | |  |
| ChIP-YUC6-5-FP | | | | acgggaaaatacaaaggctggtattatc | |  |
| ChIP-YUC6-5-RP | | | | gttggaacttgaagtcacaagggc | |  |
| ChIP-YUC6-6-FP | | | | TCCAATACAAGAGTTCCCTGAGGGA | |  |
| ChIP-YUC6-6-RP | | | | TCCATTGATGCTCCACTAATCCCA | |  |

FP, Forward primer; RP: reverse primer; LB (GABI-LEFT): GABI T-DNA left border primer
